# Differentially Expressed Genes in EEC and LMS Syndromes

**DOI:** 10.1371/journal.pone.0129432

**Published:** 2015-06-15

**Authors:** Wei Yin, Yaling Song, Yangge Du, Zhuan Bian

**Affiliations:** 1 Key Laboratory of Oral Biomedical Engineering of the Ministry of Education, Hospital and School of Stomatology, Wuhan University, Wuhan, Hubei, China; 2 Department of Endodontics & Periodontics, College of Stomatology, Dalian Medical University, Dalian, Liaoning, China; 3 School of Stomatology, Wuhan University, Wuhan, Hubei, China; NIDCR/NIH, UNITED STATES

## Abstract

**Objectives:**

Ectrodactyly ectodermal dysplasia cleft lip/palate (EEC) syndrome and limb-mammary syndrome (LMS) share a similar phenotype and the same pathogenic gene, which complicates the ability to distinguish between these diagnoses. The current study aims to identify a potential and practical clinical biomarker to distinguish EEC from LMS.

**Methods:**

Two EEC pedigrees and one LMS pedigree that have been previously reported were reanalyzed. After confirmation of the causative mutations for these new patients, whole-genome expression microarray analysis was performed to assess the molecular genetic changes in these families.

**Results:**

Five new patients with classic symptoms were reported, and these individuals exhibited the same mutation as their relatives (c.812 G>C; c.611G>A; and c.680G>A). According to the whole genome expression results, the EEC patients exhibited different gene expression characteristics compared with the LMS patients. More than 5,000 genes were differentially expressed (changes >2 or <0.5-fold) among the EEC patients, LMS patients and healthy individuals. The top three altered pathways have been implicated in apoptosis, the hematopoietic cell lineage and the Toll-like receptor signaling pathway.

**Conclusion:**

Our results provide additional clinical and molecular information regarding EEC and LMS and suggest that peripheral blood cytokines may represent a promising clinical biomarker for the diagnosis of these syndromes.

## Introduction

Ectrodactyly ectodermal dysplasia cleft lip/palate (EEC) syndrome (#OMIM 604292), which is characterized by split hands and feet, ectodermal dysplasia, and cleft lip and/or cleft palate, is a rare inherited condition. EEC shares clinical features with acro-dermato-ungual-lacrimal-tooth (ADULT) syndrome (#OMIM 103285), ankyloblepharon-ectodermal dysplasia-clefting (AEC) syndrome (#OMIM 106260), limb-mammary syndrome (LMS) (#OMIM 603543), orofacial cleft 8 (#OMIM 129400), and Rapp-Hodgkin ectodermal dysplasia (RHS) (#OMIM 129400). Increasing numbers of publications have recently reported cases with overlapping features shared among these syndromes [1–4]. The phenotypic spectrum and variable expressivity make the clinical diagnosis and classification of these syndromes difficult [5].

Moreover, these syndromes are caused by mutations in the same gene, i.e., the tumor prot*ein p63 (TP63)* gene [6]. The TP63 gene is a key regulator of ectodermal, orofacial and limb development. It is located at 3q28 and has 15 exons. Multiple transcript variants of the TP63 gene, which involve alternative splicing and different promoters, encode six isoforms, and the full-length TP63 protein contains 448 amino acids [7].

Based on the similar phenotypes and the same causative gene, some reports [5, 8–13] have proposed the use of the term “TP63 syndromes” for these related disorders, which has further perpetuated the confusion among clinicians in the differentiation of these syndromes.

This study was designed to determine an operable method for clinicians to arrive at a diagnosis. Two EEC pedigrees and one LMS pedigree that we reported in 2010 [14] and five new patients were analyzed. After confirming the causative mutations in these new patients, we used a whole-genome expression microarray to investigate the molecular genetic changes in these families and sought to determine peripheral blood biomarkers for EEC and LMS, respectively.

## Materials and Methods

### Ethical approval

This study was approved by the Institutional Review Board (IRB) of the Hospital and School of Stomatology, Wuhan University. Written informed consent was obtained from all participants or their guardians. The individuals in this manuscript provided written informed consent (as outlined in the *PLOS* consent form) to publish these case details.

### Participants, sample collection and mutation screening

These three pedigrees have been previously reported [14]. During the reanalysis from August to October 2011, we encountered five new patients (one EEC patient in pedigree I, one EEC patient in pedigree II and three LMS patients in pedigree III). The diagnosis, clinical examination, venous blood sample collection, DNA extraction and mutation detection were performed as described in the previous report [14]. Each participant underwent a general physical examination to confirm their condition prior to sample extraction.

### Human long oligonucleotide microarray

The human genome 70-mer oligonucleotide microarray was obtained from CapitalBio Corporation (Beijing, China). A total of 21,329 well-characterized *Homo sapiens* genes were included in the microarray. Eight subjects, which comprised two EEC patients, two LMS patients and four healthy controls, were recruited for this study.

### RNA extraction, amplification, labeling and hybridization

RNA was extracted and purified using the TRIZOL reagent (Invitrogen, Gaithersburg, MD, USA) and the RNeasy Mini Kit (Qiagen, Valencia, CA, USA) following the manufacturers’ instructions. RNA agarose gel electrophoresis was used to assess the RNA quality. cDNA labeled with a fluorescent dye (Cy5 and Cy3-dCTP) was produced using Eberwine’s linear RNA amplification method and subsequent enzymatic reaction, the details of which have been previously reported [15].

### Microarray imaging and data analysis

A confocal scanner and software (both obtained from CapitalBio, Beijing, China) were used to scan and analyze the images. Faint spots were removed to extract the individual channel data. The spots with intensities under 400 units, following background subtraction in the Cy3 and Cy5 channels, were considered faint. The LOWESS program was used to normalize the data.

### Definition of differentially expressed genes

The criteria for determining the significance of differentially expressed (over- or under-expressed) genes included a normalized intensity ratio greater than two-fold and a ratio of less than one half. Significance Analysis of Microarrays (SAM&R) software and T-tests were also performed for each gene. Genes with non-significant P values (P > 0.05) were excluded. GoMiner software (http://discover.nci.nih.gov/gominer/) was used to analyze the functions of these differentially expressed genes.

### Confirmation of microarray data by real-time polymerase chain reaction (PCR)

To verify the microarray analysis results, the significantly down- and up-regulated genes were validated using real-time PCR, which was performed with the Platinum Quantitative RT-PCR One-Step System (Invitrogen, Carlsbad, CA, USA) following the manufacturer’s instructions. The housekeeping gene glyceraldehyde 3-phosphate dehydrogenase (GAPDH) was used as a control.

Relative quantification was used to analyze the results of the RT-qPCR analysis. Differences in the fold change were calculated using the 2^–ΔΔCT^ method (2^–ΔΔCT^ > 2 indicates an increase, whereas 2^–ΔΔCT^ < 0.5 indicates a decrease). T-tests were performed (SPSS 13.0 for Windows, SPSS, Chicago, IL, USA) to identify statistically significant differences. A P value < 0.05 was considered significant.

## Results

### Clinical findings and mutation analysis

Each of the five new patients was of Han descent and lived in the middle region of China. These subjects ranged in age from teenagers to adults in their fifties. The two new EEC patients, one male and one female, exhibited classic features that included ectodermal dysplasia, ectrodactyly, and orofacial clefts. The three new LMS patients, two males and one female, exhibited severe limb abnormalities. The 25-year-old female also had nipple anomalies similar to the proband. The other family members were clinically healthy. These new patients exhibited the same causative mutations as their relatives (c. 812 G>C; c.611G>A; and c.680G>A).

### Gene expression profiling

**Fig 1:**
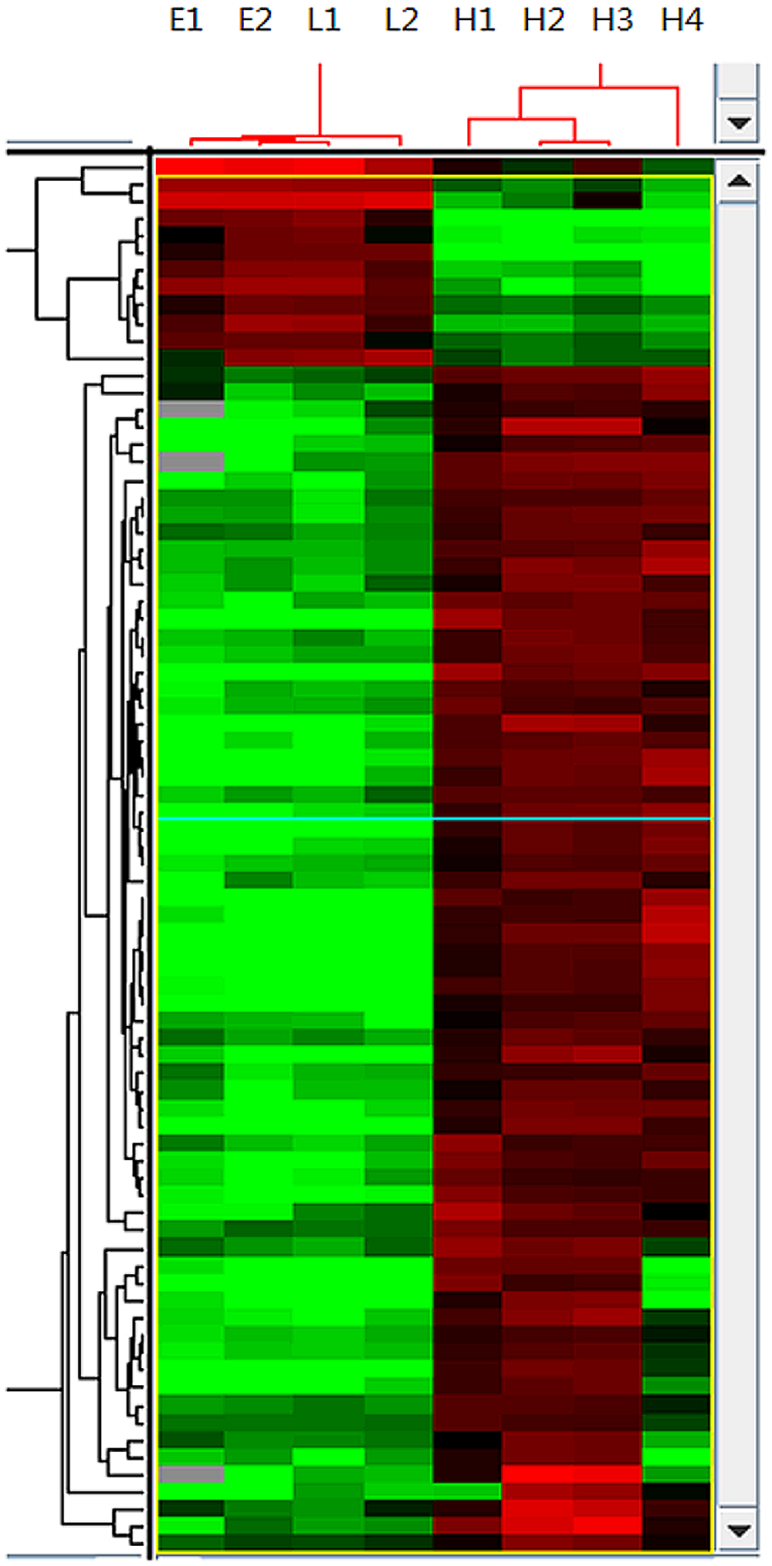
Hierarchical clustering of gene expression profiles for the eight samples. The color in each well represents the relative expression of each gene (vertical axis) in each sample (horizontal axis). Red: up-regulated genes; green: down-regulated genes. E: patients from the EEC pedigree; L: patients from the LMS pedigree; H: healthy controls.

**Fig 2:**
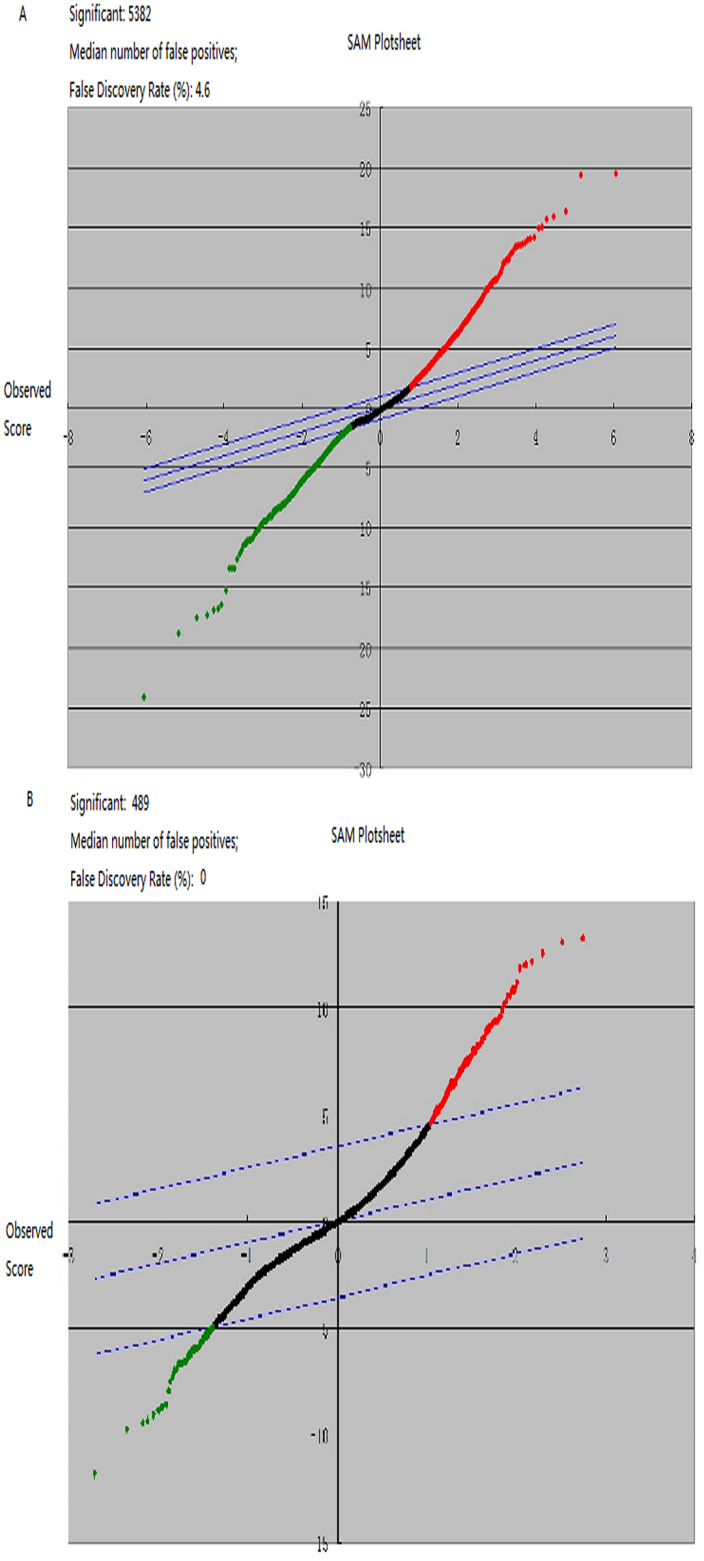
Clustering results of differentially expressed genes. (a) When the criteria was changes >2 or <1/2, 5,382 genes were differentially expressed among EEC patients, LMS patients and healthy controls. (b) When the criteria was changes >3 or <1/3, 5,382 genes were differentially expressed. The red spots represent up-regulated genes, whereas the green spots represent down-regulated genes.

More than 5,000 genes were differentially expressed among the EEC patients, LMS patients and healthy individuals (Figs 1 and 2a). The top three altered pathways included the apoptosis pathway, the hematopoietic cell lineage and the Toll-like receptor signaling pathway.

**Fig 3:**
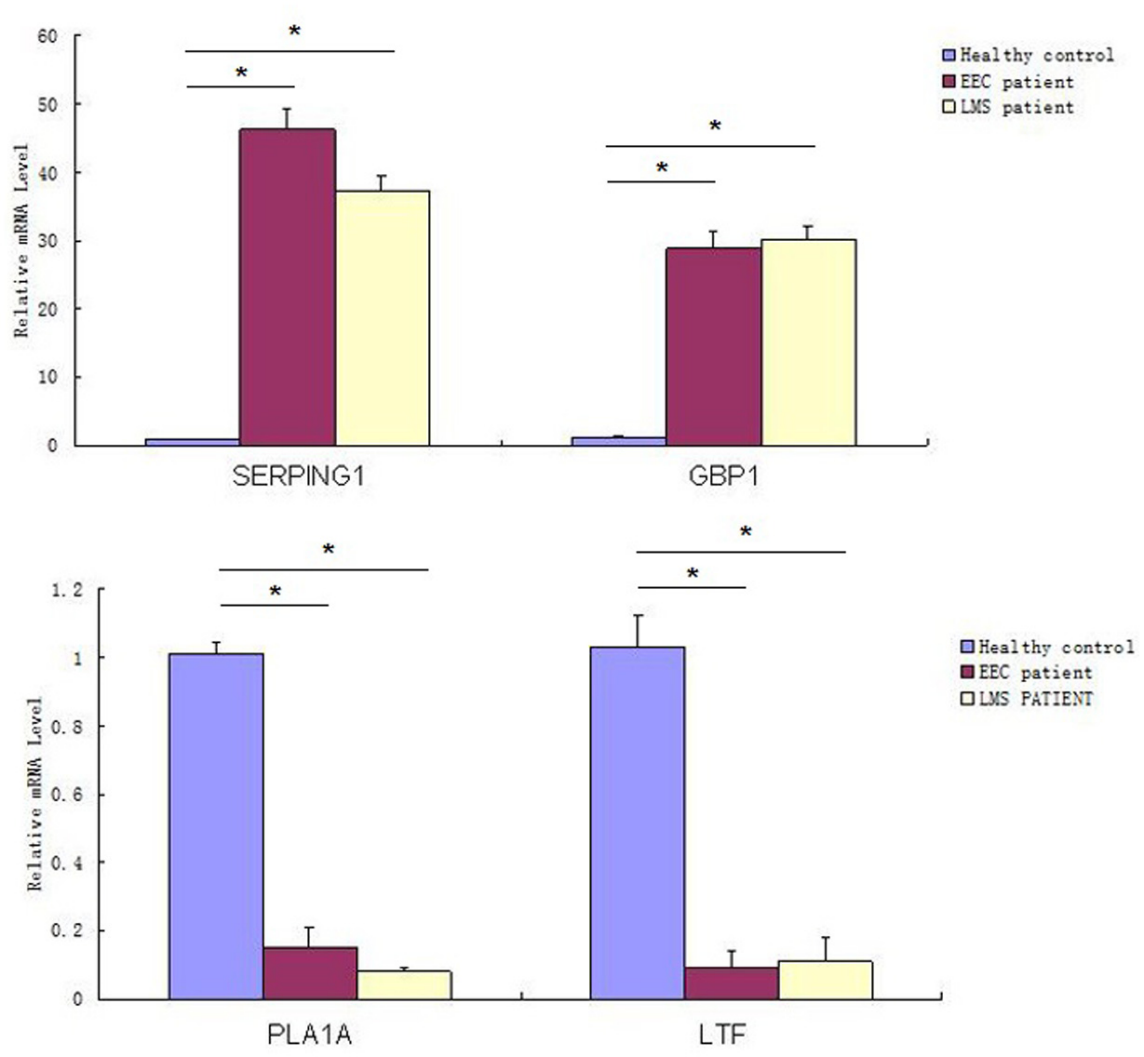
SERPING1, GBP1, PLA1A and LTF gene mRNA expression. (*:p<0.01;**:P<0.05).

**Fig 4:**
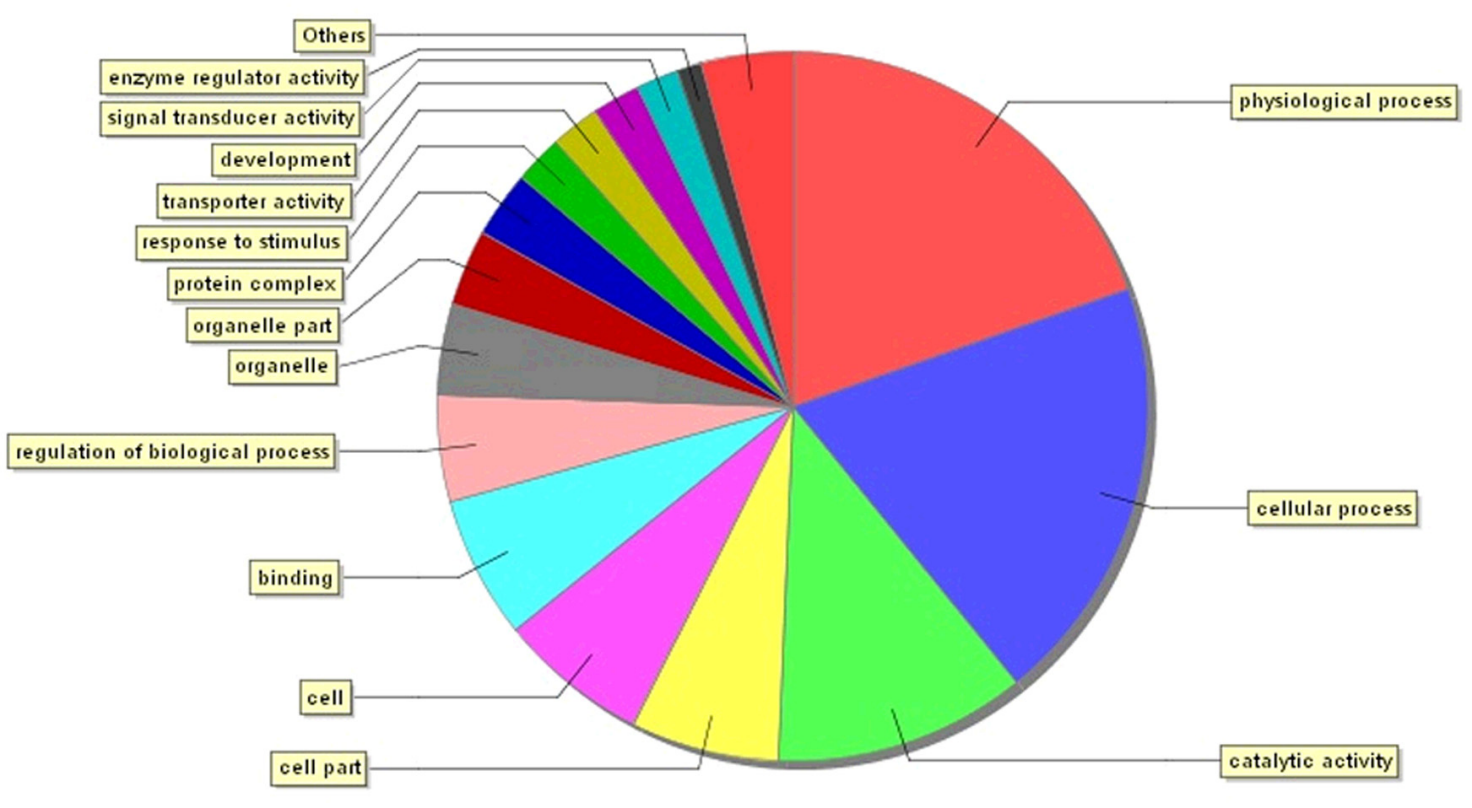
Functional analysis of genes that exhibited altered expression levels.

Between the EEC patients and LMS patients, 2,064 genes were altered genes, with 1,126 genes up-regulated and 938 genes down-regulated. If we narrowed the selection criteria of the significant differentially expressed genes to more than three-fold or less than one third, 489 genes belonged to this scope (Fig 2b). The top three related pathways included the hematopoietic cell lineage, cytokine-cytokine receptor interaction and MAPK signaling pathways. The top five up-regulated genes included genes that encode the serine/cysteine proteinase inhibitor clade G (C1 inhibitor) member 1 (SERPING1); guanylate binding protein 1 (GBP1); purinergic receptor P2Y, G-protein coupled, 14 (P2RY14); tumor necrosis factor alpha-induced protein 6 (TNFAIP6); and guanylate binding protein 3 (GBP3). In contrast, the top five down-regulated genes were phospholipase A1 member A (PLA1A), lactotransferrin (LTF), adenylate kinase 3 (AK3), stromal antigen 3 (STAG3) and polymeric immunoglobulin receptor (PIGR). These altered genes exhibited expression changes of approximately five- to ten-fold. All these obviously changed genes were confirmed via real-time PCR. Except the GBP1 and LTF gene, others were identical (Fig 3). Half of these altered genes participated in cellular processes, physiological processes and catalytic activity (Fig 4).

## Discussion

The phenotypic features of the previously discussed TP63 mutation-related syndromes are quite similar, especially for AEC and RHS, as well as EEC and LMS. Nevertheless, these syndromes can be distinguished. Ectodermal dysplasia, split hands and feet, and a cleft palate with or without cleft lip are the three main EEC syndrome features. AEC is characterized by ankyloblepharon filiforme adnatum, cleft palate, ankyloblepharon, and ectodermal defects and also exhibits congenital adhesions between the upper and lower jaws. RHS exhibits characteristic midfacial hypoplasia and ectodermal dysplasia, as well as a cleft lip and palate. LMS syndrome is characterized by severe hand/foot anomalies and hypoplasia/aplasia of the mammary gland and nipple. ADULT presents with ectrodactyly, mammary hypoplasia, and excessive freckling without facial clefting.

In clinical practice, it is difficult to correctly diagnose these syndromes. First, the highly variable expression and reduced penetrance, which results in marked intra-familial and inter-familial variability, make the situation complex. For example, most EEC patients do not manifest all three cardinal abnormalities. Approximately one-half of patients develop abnormalities of the hair, skin, teeth or nails; however, only one-third of patients exhibit skin abnormalities. Approximately 70% of patients exhibit ectrodactyly, whereas only 43% of patients exhibit syndactyly. Two-fifths of patients presented with a cleft lip or palate [1,2], and other syndromes have also exhibited similar trends. The incomplete penetrance interferes with a correct clinical diagnosis.

Second, these syndromes have the same causative gene, *TP63*. TP63 has been identified in a variety of human and mouse tissues, including proliferating basal cells of the epithelial layers in the epidermis, and this protein exhibits strong homology to the tumor suppressor TP53. The full-length TP63 consists of six domains: 1) the transactivation (TA) domain, 2) the DNA binding domain (DBD), 3) the tetramerization (ISO) domain, 4) the second transactivation (TA2) domain, 5) the sterile-a-motif (SAM), and 6) the transactivation inhibitory domain (TID) [16]. There have been strong genotype–phenotype correlations in some of these syndromes. Mutations of AEC and RHS syndromes, which are inclined to cluster in the SAM and TID domain of the TP63 gene, primarily comprised missense and frameshift mutations. Although a correlation was not identified in the other syndromes, mutations in DBD have been identified in several syndromes. Therefore, DNA sequence analysis cannot aid clinicians in distinguishing between these syndromes.

**Fig 5:**
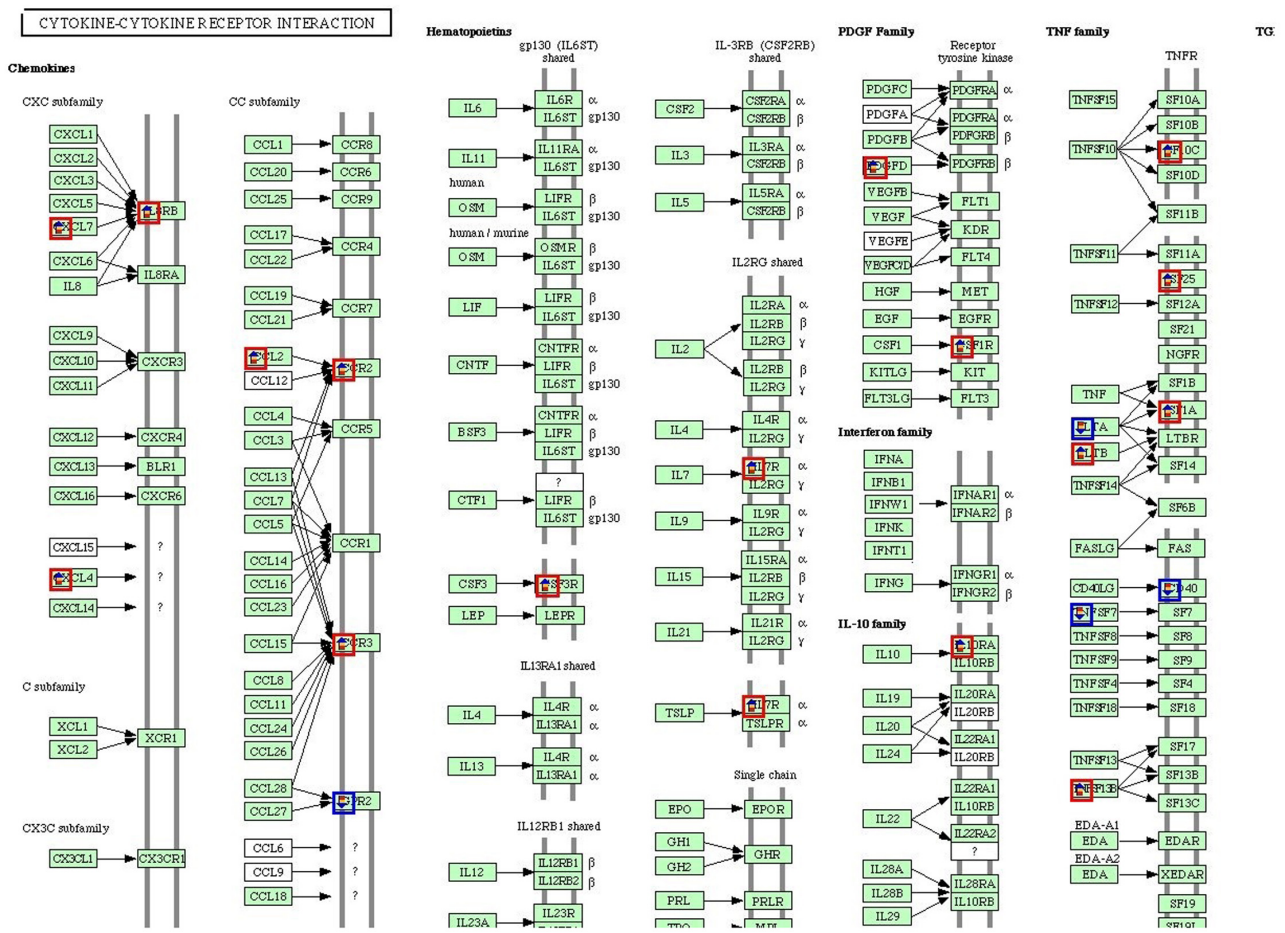
Differentially expressed genes among these three groups were involved in the inflammation signal pathway. Red and blue represents the up- and down- regulated genes respectively.

To identify a potential practical clinical biomarker for EEC syndrome and LMS syndrome, we investigated the gene expression characteristics of these patients. The data suggested that the molecular mechanisms responsible for EEC and LMS were different, with numerous genes differentially expressed between these syndromes. More than 2,000 genes were differentially expressed by at least two-fold. Furthermore, 489 genes remained changed even if the selection criteria were narrowed to three-fold. Forty-three of these 489 genes were related to immune and inflammatory responses (Fig 5). Four (SERPING1, GBP1, P2RY14 and GBP3) of the top five up-regulated genes were related to the immune response, whereas SERPING1 and TNFAIP6 were related to inflammation. For the molecular function analysis, most genes were related to receptor activity, sugar binding and calcium ion binding.

TP63 and its isoforms are over-expressed in a wide variety of human malignancies, such as cervical, head and neck, and lung cancer [17]. Furthermore, deletions, mutations and interactions with cellular or viral proteins have been recognized as key steps in the development of approximately half of all human cancers. Moreover, the TAp63 isoform had p53-like functions, whereas DNp63 functioned as a dominant negative inhibitor of the p53 family [18,19], which raises the question as to whether patients with TP63 mutations have an increased tumor incidence. However, there was no increased tumor incidence in our patients. Whole-genome gene expression analysis indicated that apoptosis pathway expression was significantly increased in the patients with TP63 mutations compared with the healthy controls.

Unfortunately, the limitation of this study was evident. Only four TP63 mutation patients were identified. Data from large patient samples are necessary in future investigations.

In conclusion, the gene expression profiles of these Chinese EEC and LMS patients with TP63 mutations were significantly different. Our findings suggest that inflammatory cytokines in peripheral blood may represent potential biomarkers for the clinical diagnosis of these syndromes. Additional studies regarding other TP63 mutation syndrome patients are needed to verify this conclusion.
